# An experimental study on the influence of healthy physical education curriculum model on sports ability of Chinese senior high school students

**DOI:** 10.1371/journal.pone.0298858

**Published:** 2024-05-14

**Authors:** Shengting Dai, Qian Qiu, Yuancai Zhang, Jingfei Yan, Rongbin Yin

**Affiliations:** 1 School of Sports Science and Engineering, East China University of Science and Technology, Shanghai, China; 2 Physical Education College, Xuchang University, Xuchang, China; 3 College of Physical Education and Health, East China Normal University, Shanghai, China; 4 Ministry of Physical Education, Shanghai Institute of Technology, Shanghai, China; 5 School of Physical Education, Soochow University, Suzhou, China; GSVM Medical College, INDIA

## Abstract

In recent years, the growing incidence of health issues among Chinese students, including obesity, diabetes, and other chronic diseases, has been attributed to a sedentary lifestyle, lack of physical activity, and unhealthy eating habits. Physical education (PE) classes play a crucial role in promoting physical activity and fostering healthy lifestyles among Chinese students. The purpose of this study was to investigate the influence of the healthy PE curriculum model on the sports ability of senior high school students in China. The trial adopted a quasi-experimental design with equivalent groups. The experimental group followed the healthy PE curriculum model in their PE classes, while the control group received traditional technical instruction. During the 12-week intervention, 149 senior high school students completed the sports ability test as both the pre-test and post-test measurements for this experimental study. The results indicated that the experimental group showed significant improvements in sports ability compared to the control group, highlighting the positive effects of the healthy PE curriculum model. The structural characteristics of the healthy PE curriculum model provided essential support for students’ learning and proved to be an effective way to promote physical literacy among senior high school students in China.

## 1. Introduction

Physical education plays a crucial role in the development and well-being of students, especially during their formative years in senior high school [1, 2]. With the growing concern for the sedentary lifestyles and poor health habits of today’s youth, there is a need for innovative physical education models that can help promote healthy lifestyles and enhance sports abilities [3]. In recent years, China has witnessed a dramatic increase in the number of students who suffer from health issues due to lack of physical activity [4, 5]. The traditional physical education curriculum model in China is exam-oriented, which means that students focus mainly on achieving good grades rather than improving their physical fitness [6]. Therefore, there is a need to evaluate the effectiveness of physical education curricula in promoting physical activity and improving sports ability among Chinese senior high school students [7].

To effectively advance the implementation of China’s new primary and secondary school curriculum standards and foster the development of students’ core competencies in physical education and health, Professor Ji Liu, who led the 2015 National Primary and Secondary School Physical Education and Health Curriculum Standards Development and Revision Group and is associated with East China Normal University, drew inspiration from the” National Medium and Long-term Education Reform and Development Plan (2010–2020).”Guided by the fundamental principles outlined in the national “Physical Education and Health Curriculum Standards” for primary and secondary schools, Professor Ji Liu amalgamated international insights from physical education curriculum models and domestic best practices. This culminated in the proposal of a comprehensive physical education and health curriculum model, aptly named “The Healthy Physical Education Curriculum Model(HPECM),which harmoniously blends an international perspective with distinctively Chinese characteristics [8]. HPECM (Table 1) plays a pivotal role in driving the implementation of the national”Curriculum Standards” in the domain of physical education and health. It not only aligns with the principles and ethos of the “Curriculum Standards” but also offers clear guidance and methodologies to address critical issues in curriculum development, as articulated by Professor Ji Liu in 2015.

**Table 1:** Comparison of two teaching model designs.

Model	Preparatory Part (15 minutes)	Basic Part (60 minutes)	End Part (5 minutes)	Exercise Density	Exercise Intensity
• The Healthy Physical Education Curriculum Model	• Classroom routine • General preparation activities and specific preparation activities • Combining movement and stillness with music • Lively atmosphere • Stimulate interest • Use gamification to make every student happy and active	• Motor skill learning (Approximately 45minutes): Primarily focused on activities and games • Physical fitness: (Approximately 15 minutes) Emphasis on diversity, fun, and compensation	• Combined with music and other means to carry out diverse relaxation exercises • Teachers and students jointly summarize or evaluate	• The exercise density is above 75%	• exercise intensity is 140–160 times/min
• Traditional Physical Education Curriculum Model	• Classroom routine • Jogging • Freehand exercises or joint movement exercises	• Teacher explains and demonstrates • student practice • Teacher guidance	• Relaxation with bare hands as the main form • Teacher summary	• The exercise density is above 30%	• exercise intensity is less than 140

This model adopts a comprehensive curriculum perspective, operating within the framework of curriculum discourse. It encompasses the holistic design of the curriculum, ranging from modules to individual units, with a strong emphasis on effective curriculum implementation. This approach outlines overarching requirements and specific implementation strategies."

The main purpose of the Healthy Physical Education Curriculum Model is to solve the problem of the continuous decline in the physical health level of Chinese teenagers in the past 30 years. The implementation of the Healthy Physical Education Curriculum Model requires grasping three key points: sports skills, physical fitness, and exercise load. In terms of sports skills, structured skill learning and practice are emphasized. Whether it is a new teaching or a review class, attention should be paid to activities and competitions, and single-skill teaching should be rejected, with time maintained at around 20 minutes (a 40-minute class as an example). In terms of physical fitness, diversified, interesting, and compensatory physical fitness design is emphasized, with time maintained at around 10 minutes. In terms of exercise load, it is emphasized that the average heart rate of students in the classroom should reach 140–160 times per minute, and the continuous exercise time of students should account for about 75% of the total class time. It should be noted that the exercise time of each student does not need to reach 75% of the total class time, but the teacher should arrange the entire activity time to not exceed 25% of the total class time in a static state. The structural characteristics of the Healthy Physical Education Curriculum Model not only help improve students’ sports ability but also contribute to the cultivation of students’ physical literacy [9].

Physical education curriculum models have been developed to address this issue and improve the quality of PE programs. Several studies have examined the effectiveness of the HPECM in improving sports ability outcomes among youth [10, 11]. However, most of these studies have focused on elementary and middle school students. There is a need for research on the influence of the HPECM on sports ability among senior high school students. Therefore, the aim of this experimental study is to investigate the influence of HPECM on the sports ability of Chinese senior high school students. The study seeks to evaluate whether a curriculum model that emphasizes healthy physical activity can improve the sports ability of students and promote a more active lifestyle.

## 2. Materials and methods

### 2.1 Study design

Before the experimental research, the teacher of the experimental group was trained in the theory and practice of the healthy physical education curriculum model and achieved satisfactory training results. According to the school facilities, teachers’ characteristics, and students’ learning experience, the teaching experiment theme is basketball. Basketball teaching lasts for 12 weeks, two classes per week, for a total of 24 classes, each class lasting 80 minutes.

The experimental group firmly grasped the three key points of the healthy physical education curriculum model, and the teaching plan was jointly written between researcher and teacher after discussion. The control group implements routine teaching [12], that is, technical-traditional teaching, which has the following remarkable characteristics: The average heart rate of students in the whole class is less than 140 beats/min; Mainly adopts single movement skill teaching; There is no special physical exercise in the class. During the 12-week intervention period, all students completed the test of sports ability variables two times as the pre-test and post-test of this experimental study.

During the experiment, it is important to minimize the influence of irrelevant factors on the results. The control process mainly involves the following aspects: using identical teaching materials for both groups; ensuring that the teaching time is the same for both groups; adjusting the amount of teaching based on the weather and various school activities that may affect the experiment; to reduce experimental errors caused by differences in teaching styles and abilities among different teachers, the experimental and control groups are taught by the same teacher, and the experimental teacher is instructed to strictly follow the experimental plan while the control group should not intentionally imitate the experimental group’s teaching. Both the experimental and control groups were the students’ usual teachers. The principal investigator is present to assist and correct any problems that arise to ensure the effectiveness of the experimental teaching in the experimental group.

**Inclusion/exclusion criteria**:

**Inclusion Criteria**:

Educational Level: Participants must be currently enrolled high school students attending physical education classes.

Grade Range: Participants must specifically be high school sophomores.

Prior Physical Education Experience: Eligible participants should have successfully completed at least one year of physical education coursework.

Consent: Prior to participation, written informed consent is mandatory from both the students and their legal guardians.

**Exclusion Criteria**:

Medical Conditions: Participants with documented cardiovascular or respiratory conditions that may be adversely affected by physical activity will be excluded from the study.

Advanced Knowledge of Experimental Methods: Students possessing prior exposure or training in the experimental teaching methods under investigation will be excluded from participation.

These criteria aim to ensure a clear definition of the study’s target population, emphasizing the requisite educational level, grade, and prior experience in physical education. The inclusion of specific exclusion criteria serves to safeguard participant safety and maintain the integrity of the experimental design. Obtaining written informed consent further underscores the ethical considerations of the study.

**Intervention condition and the control condition**:

**Intervention Condition:** The intervention condition centers on the application of The Healthy Physical Education Curriculum Model (HPECM). Grounded in contemporary pedagogical theories, HPECM seamlessly integrates cutting-edge teaching strategies tailored to elevate student engagement, facilitate skill acquisition, and optimize overall outcomes in physical education. This intervention marks a departure from conventional didactic methods, introducing a student-centric paradigm. HPECM adopts an innovative approach by incorporating technology-assisted learning tools, personalized feedback mechanisms, and collaborative learning experiences. The overarching objective is to create an enriched educational environment that transcends traditional instructional practices.

**Control Condition:** To mitigate potential experimental errors arising from variations in physical education teaching methods, basketball is consistently chosen as the focal subject for this study. In addressing potential confounding factors linked to diverse teaching styles and instructor capabilities, a meticulous control strategy is implemented. Specifically, both the experimental and control classes within the same academic period are instructed by the same teacher. To ensure consistency, explicit instructions are provided to the experimental teachers, mandating adherence to the experimental plan. Conversely, the control class is advised against intentionally mimicking the experimental conditions. Throughout the teaching sessions, the experimental instructor actively engages in problem-solving, offering assistance and corrections promptly.

### 2.2 Participants

The research study selected second-year students from a high school in Shanghai as research objects. Class selection was conducted through a randomization procedure. A random number generator was employed to allocate classes to either the experimental or control group. This approach aimed to ensure an unbiased selection process and create comparable groups at the study’s outset. Two classes from the second grade of high school were randomly selected as Experimental Group 1 (girls class) and Experimental Group 2 (boys class) due to the influence of class division teaching. Additionally, two classes were randomly selected as Control Group 1 (girls class) and Control Group 2 (boys class). Students were asked about their past medical history, family genetics, cardiovascular disease status, etc., to rule out potential risks in sports. There were 76 students in the experimental group (36 boys and 40 girls), and 73 students in the control group (35 boys and 38 girls). Please refer to Table 2 for the mean and standard deviation of the age, height, and weight of students in each experimental class and control class. During the process of recruiting subjects, there were no refusals to participate, and all subjects participated fully in the teaching experiment.

**Table 2:** Basic information of experimental group and control group students.

	Experimental Group(N = 76)		Control Group(N = 73)	
Gender	**Boys(N = 36)**	**Girls(N = 40)**	**Boys(N = 35)**	**Girls(N = 38)**
Age	16.47±0.56	16.5±0.56	16.23±0.49	16.26±0.50
Height(cm)	178.00±5.94	164.20±5.35	175.54±4.76	164.45±4.92
Weight (kg)	72.89±12.66	56.2±7.92	69.1±10.48	58.21±8.36

### 2.3 Ethics statement

Prior to conducting the study, ethical approval was granted by the University Committee on Human Research Protection (UCHRP) of East China Normal University. The research protocol, including the participant consent process, was reviewed and approved by the UCHRP under protocol number [Approval Number: HR 095–2019].

All participants in this study were voluntary respondents selected by the research panel. They were provided with detailed information about the study’s purpose, procedures, potential risks, and benefits. Participants were assured that their responses would be treated as strictly confidential and anonymous. The confidentiality of their data was maintained throughout the research process. Participants provided written informed consent to participate in this study. In accordance with the approved protocol, each participant confirmed their willingness to participate by endorsing the following statement on a paper questionnaire: "I voluntarily consent to participate in this research project. I understand that my participation is entirely voluntary and that I have the right to withdraw from the study at any point without any negative consequences."

### 2.4 Data collection

Data were collected over a period of 3 months (From October 2020 to December 2020), incorporating pre-test and post-test measurements. The data collection process comprised the following steps:

Pre-Test Assessment: Prior to the intervention, baseline measurements were obtained to assess the initial sports ability level of the participants.

Post-Test Assessment: Following the intervention period, post-test measurements were conducted to evaluate the impact of the healthy physical education curriculum model. The same assessment components used in the pre-test were administered to both the control and intervention groups. The post-test assessments use the same standardized protocols and equipment as the pre-test.

### 2.5 Experimental intervention program

#### 2.5.1 Motor skill intervention program

The motor skill exercises for both the experimental and control groups were standardized to focus on basketball. This design was implemented to eliminate potential interference from varying motor skill exercises on the experimental results [13–16]. The design of motor skill instruction in the experimental group adhered to the guidelines set forth by the HPECM for motor skill teaching. Structured basketball skill instruction was employed to empower students to master a comprehensive set of motor skills. The approach advocated against the exclusive teaching of isolated techniques, instead emphasizing theintegration of basketball learning within practical activities and competetive scenarios. During basketball instruction, a priority was placed on minimizeing breaks, optimizing group dynamics, and enabling students to engage in supplementary exercises during idle moments. Teachers also reinforced precise movement techniques, encouraged cooperative practice, and instilled a sense of courage and initiative.

#### 2.5.2 Physical fitness intervention program

The principles governing physical fitness program design were aligned with the requirments of the HPECM. These princples were thougfully talored to suit the unique physical and psychological characteristics of high school students:

1. Emphasis was placed on diversification and enjoyment in the methods and approaches employed for physical fitness exercises. This approach aimed to counteract the monotony often associated with endurance focused activities like running laps, repetitive exercises such as sit-ups for core strength, and sprinting for speed.
2. Special attention was given to the concept of "compensatory" physical fitness exercises. Considering the distinctive demands of basketball within this study, students were encouraged to participate in supplementary exercises targeting core abdominal strength, cardiovascular endurance, and agility. This approach was integrated into the basketball curriculum, fostering a holistic and balanced approach to physical fitness development among students.
3. Efforts were made to avoid transforming physical education classes into mere physical fitness testing sessions.

#### 2.5.3 Motor cognitive ability intervention program

The intervention principles of the motor cognitive ability intervention plan were formulated in accordance with the spirit of the HPECM and the contemporary teaching philosophy. These principles were strategically designed to accommodate the physical and psychological development characteristics inherent to high school students. Within the classroom setting, students were introduced to motor cognitive ability related knowledge through engaging PowerPoint presentations. Experimental teachers placed a strong emphasis on cultivating student’s motor cognitive ability throughout the teaching process. For instance, during warm-up exercises, teachers underscored the critical role of preparation activities in injury prevention and highlighted the benefits of relaxation in alleviating exercise-related fatigue.

### 2.6 Test variables

The assessment of sports ability includes three aspects: motor skills, physical fitness, and motor cognitive ability. Motor skills mainly include single skill assessment, combination skill assessment, and performance assessment in competition. Physical fitness tests mainly include indicators such as body composition, muscle strength, muscle endurance, Cardiopulmonary function, speed, flexibility, and agility. Motor cognitive ability is mainly assessed using scale tests. See Table 3 for specific details.

**Table 3:** Senior high school student sports ability test content table.

Indicators	Specific Test Content
**Motor Skill**	Basketball single skill; basketball combination technique/skill; basketball game performance
**Physical Fitness**	Body composition: body fat percentage、waist-to-hip ratio; Muscular strength: grip strength、Standing long jump Muscular endurance:1-minute crunches Cardiopulmonary function: vital capacity、20-meter shuttle run Flexibility: seated forward bend Speed:50-meter dash
**Motor Cognitive**	Agility:20-second lateral shuffles
**Ability**	Motor cognition、physical fitness awareness

#### 2.6.1 Motor skill test

Testing Protocol:

Single Techniques: Participants are assessed on fundamental basketball skills, including shooting. Each participant is given a specific task, and their execution is observed and rated based on established criteria.

Combination Techniques: This segment evaluates participants’ ability to seamlessly integrate various basketball skills. The task is passing and shooting, assessing the fluidity and effectiveness of their execution.

Match Performance: Participants engage in a simulated basketball game, applying their skills in a dynamic and competitive context. This allows for the assessment of strategic decision-making, teamwork, and adaptability during actual gameplay.

Evaluation Method:

Three experienced teachers serve as evaluators for each test, ensuring a comprehensive and reliable assessment. Each teacher scores participants independently based on predefined criteria for each skill and performance aspect. The final score for each participant is derived by calculating the average of the three individual scores.

Scoring Criteria:

1.Single Techniques

[85 points—100 points] Standard Attainment (70%): 6 males, 4 females; Technical Evaluation (30%): Demonstrates accurate posture and distinct finger flicking motion.

[75 points—84 points] Standard Attainment (70%): 4 males, 3 females; Technical Evaluation (30%): Exhibits relatively accurate posture with noticeable finger flicking motion.

[60 points—74 points] Standard Attainment (70%): 3 males, 2 females; Technical Evaluation (30%): Displays accurate posture with no discernible finger flicking motion.

[59 points and below] Standard Attainment (70%): 2 males, 1 female; Technical Evaluation (30%): Features inaccurate posture with no discernible finger flicking motion.

2.Combination Techniques

[85 points—100 points] Exceptional proficiency in passing and dribbling, demonstrating accurate passing, a correct and effortless shooting motion, and seamless coordination in passing, receiving, and shooting. Males complete the task within 45 seconds, and females within 56 seconds, with a successful shot count of 3 or more.

[75 points—84 points] Competent in passing and dribbling, showcasing correct shooting form and excellent coordination. Males complete the task within 50 seconds, and females within 60 seconds, with a successful shot count of 2 or more.

[60 points—74 points] Adequate execution of passing and shooting, albeit with slightly weaker coordination. Males complete the task within 55 seconds, and females within 65 seconds, with a successful shot count of 1 or more.

[59 points and below] Notable errors and significant mistakes in passing and shooting. Males complete the task in 55 seconds or more, and females in 65 seconds or more, with a successful shot count of 0.

3. Match Performance

[85 points—100 points]Personal Attack Ability: Proficient utilization of offensive skills (shooting, penetration, break, pass, and catch the ball) with a rational and skilled approach.Defensive Ability: Demonstrates strength in individual defense and cooperative defense skills.Tactical Awareness: Exhibits a robust individual tactical action ability, quick transition between attack and defense, and a keen sense of fast attack throughout the entire game.

[75 points—84 points]Personal Attack Ability: Displays a reasonable and skilled application of offensive techniques (throwing, sudden moves, passing).Defensive Ability: Strong performance in individual defense and cooperative defense. Tactical Awareness: Possesses a strong individual tactical action ability, fast attack and defense transition speed, and a heightened consciousness of fast attacks throughout the game.

[60 points—74 points]Personal Attack Ability: Demonstrates a general level of rationality and proficiency in utilizing offensive techniques (throwing, leaping, passing).Defensive Ability: Displays average individual defense and coordination defense skills. Tactical Awareness: Possesses a moderate individual tactical action ability, with a general speed in attack and defense transition, and a reasonable awareness of fast attacks throughout the game.

[59 points and below]Personal Attack Ability: Shows poor and unskilled use of offensive techniques (throwing, sudden moves, passing).Defensive Ability: Exhibits poor individual defense and cooperative defense skills. Tactical Awareness: Displays weak individual tactical action ability, slow offensive and defensive transition speed, and a poor consciousness of fast attacks throughout the entire game.

#### 2.6.2 Physical fitness test

The body composition test includes two indexes: Percentage of Body Fat (PBF) and Waist-Hip Ratio (WHR). PBF is measured primarily by the Jawon body composition analyzer [17]. WHR is calculated by measuring the waist and hip circumference using a soft ruler, with measurements taken twice on each side and then averaged [18].

The cardiopulmonary function test includes two indicators: vital capacity [19] and a 20-meter shuttle run test [20]. Vital capacity is measured using an electronic spirometer. The 20-meter shuttle run test requires students to run back and forth between two lines that are 20 meters apart in time with the music. As the test progresses, the music speed increases. If a student cannot reach the endpoint within the required time twice in a row, the test is stopped, and the score is recorded as the number of completed 20-meter shuttles, with the unit being "times."

The muscle strength test includes two indicators: upper limb muscle strength, measured by grip strength [21], and lower limb muscle strength, measured by standing long jump [22]. For the grip strength test, the subject stands upright with their feet shoulder-width apart, arms hanging down, and palms facing inward. They then squeeze the inner and outer handles of a dynamometer with maximum effort, and the score is recorded in "kg" units. Each subject is measured twice, and the best score is taken. For the standing long jump test, the subject stands behind the take-off line with their feet together and jumps forward as far as possible without stepping or jumping in place. The distance from the take-off line to the nearest landing point is measured vertically, and the score is recorded in "m" units. Again, two measurements are taken, and the best score is taken.

The muscle endurance test measures a student’s ability to complete as many sit-ups as possible within one minute [23]. To start, the student lies down on a soft pad with their knees bent at a 90-degree angle and their hands interlaced behind their head. The examiner holds the student’s ankles on both sides, fixing their feet to the ground. Upon hearing the ’start’ command, the student completes the sit-up motion by pulling in their stomach and touching or crossing their elbows over their knees before returning to the starting position. One point is awarded for each successful sit-up completed. The examiner records the number of completed sit-ups in one minute, using "times" as the unit of measurement.

The indicator for the flexibility test is the sit-and-reach test [24]. Firstly, the teacher explains the test requirements and demonstrates. During the test, the subject sits with their feet flat against the vertical board of the measuring device, with their legs straight and not bent, and their arms straight and extended forward, pushing the cursor with their fingertips until they can no longer reach forward. Each subject is tested twice, and the best score is recorded in "cm" units.

The indicator for the speed test is the 50-meter run [25]. Firstly, the teacher explains the test requirements and organizes the students to conduct sufficient warm-up exercises. During the test, two people are tested in each group. The subjects stand at the starting line and begin to run when they hear the "run" command. The starter waves the flag at the same time as the command is given, and the timer starts timing when the flag moves. The stopwatch stops when the subject’s chest reaches the vertical plane of the finish line, and the score is recorded in "S (seconds)." The test requires the subjects not to false start, and any false starters will be called back for a restart.

**Fig 1:**
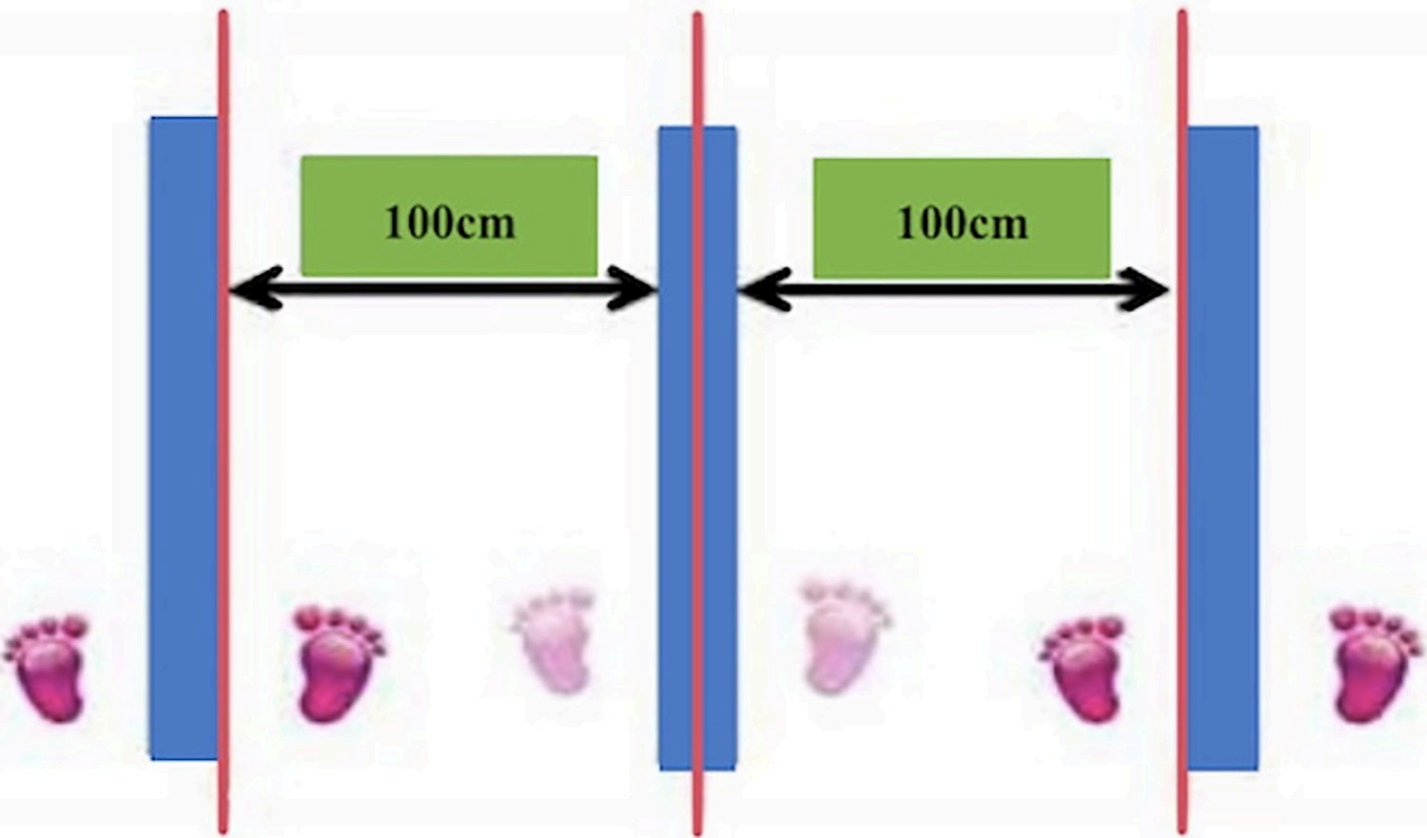
The diagram of the 20-second shuttle run.

The agility test is measured by the 20-second shuttle run [26], as illustrated in Fig 1. The subject assumes a squatting position with legs straddling the center. Upon hearing the start signal, they swiftly move to the right side and repeatedly shuttle back and forth in the order of "center→right→center→left→center…". Each time the subject crosses a line, it is counted as one, and the number of crossings completed within 20 seconds is recorded. The test is performed twice, and the best score is recorded in "times" units.

#### 2.6.3 Motor cognitive ability test

This study used a Motor Cognitive Ability Scale, which was targeted at senior high school students and included two dimensions: motor cognition and physical fitness awareness, with a total of 9 items. The "motor cognition" dimension reflected senior high school students’ knowledge and methods of sports activities, appreciation of sports, and information about sports events. The "physical fitness awareness" dimension reflected information about senior high school students’ physical development. The scale used a 5-level Likert rating, where scores from 1 to 5 were calculated based on the degree of understanding of the theme, ranging from low to high, such as "I understand the competition rules of the sports I have learned." To assess internal consistency, we calculated Cronbach’s alpha for the scale. The standardized Cronbach’s α coefficient values of the two dimensions of sports cognition and physical fitness awareness of the scale are 0.897 and 0.865 respectively, and the standardized Cronbach’s of the scale is 0.918. To assess construct validity, we conducted exploratory factor analysis (EFA) and confirmatory factor analysis (CFA).The χ2 /df for the scale is 2.839. The goodness-of-fit indces, GFI, CFI, NFI, TLI, and IFI, are 0.977, 0.987,0.980, 0.978, and 0.987, respectively. The RMSEA is 0.058, and the RMR is 0.029. All the fitting indexes met the standard of good fit and met the statistical requirements.

### 2.7 Statistical analysis

The experiment utilizes a single-factor two-group pretest-posttest design, with covariance analysis applied to process the experimental data. The pretest scores of the test indicators are treated as covariates, and the group (consisting of an experimental class and a control class) is treated as the independent variable. The posttest scores of the test indicators are used as the dependent variables. By analyzing the changes in the physical literacy-related indicators of sports and health subjects in both the experimental and control classes, the effectiveness of the experimental intervention can be determined. If there is no significant difference in the changes of the related test indicators between the two classes, a paired sample t-test is conducted to examine whether there is a significant improvement in the post-test results of each class’s related indicators.

## 3. Results

This section may be divided by subheadings. It should provide a concise and precise description of the experimental results, their interpretation, as well as the experimental conclusions that can be drawn.

### 3.1 Motor skill results

Table 4 provides descriptive statistics for the motor skill results of the experimental and control groups. The results are presented separately for each variable and by gender.

**Table 4:** Descriptive statistics for motor skill results.

Variable	Group	Gender	*N*	pre-test		post-test	
				*M*	*SD*	*M*	*SD*
1-minute shooting	Experimental Group	Girls	40	65.44	4.87	73.61	4.94
		Boys	36	76.34	12.03	80.19	11.10
	Control Group	Girls	38	67.66	5.29	73.31	6.34
		Boys	35	78.08	12.72	80.14	12.09
Passing and cutting coordination	Experimental Group	Girls	40	70.23	4.88	75.12	4.92
		Boys	36	80.92	9.58	86.31	8.96
	Control Group	Girls	38	72.13	3.79	74.39	3.43
		Boys	35	75.86	8.31	79.71	8.08
Game performance	Experimental Group	Girls	40	65.67	6.17	71.38	6.21
		Boys	36	75.31	4.94	80.61	4.47
	Control Group	Girls	38	63.55	5.70	67.68	5.02
		Boys	35	77.09	5.87	80.37	5.72

For the 1-minute shooting task, the mean scores for both girls and boys in the experimental groups increased from pre-test to post-test. The girls in the experimental group improved their mean score by 8.17 points (*SD* = 4.94), while the boys in the experimental group improved by 3.85 points (*SD* = 11.10). The control groups also saw an increase in mean scores from pre-test to post-test, with girls improving by 5.65 points (*SD* = 6.34) and boys improving by 2.06 points (*SD* = 12.09). However, the experimental group showed a larger improvement in mean scores compared to the control group. For the passing and cutting coordination task, the mean scores for both girls and boys in the experimental groups increased from pre-test to post-test. The girls in the experimental group improved their mean score by 4.89 points (*SD* = 4.92), while the boys in the experimental group improved by 5.39 points (*SD* = 8.96). The control groups also showed an increase in mean scores from pre-test to post-test, but the improvement was smaller compared to the experimental group. The girls in the control group improved their mean score by 2.26 points (*SD* = 3.43), while the boys in the control group improved by 3.85 points (*SD* = 8.08). For the game performance task, the mean scores for both girls and boys in the experimental groups increased from pre-test to post-test. The girls in the experimental group improved their mean score by 5.71 points (*SD* = 6.21), while the boys in the experimental group improved by 5.30 points (*SD* = 4.47). The control groups also showed an increase in mean scores from pre-test to post-test, but the improvement was smaller compared to the experimental group. The girls in the control group improved their mean score by 4.132 points (*SD* = 5.02), while the boys in the control group improved by 3.28 points (*SD* = 5.72).

Overall, the experimental groups showed greater improvement in mean scores from pre-test to post-test compared to the control groups across all three tasks. The results suggest that the intervention had a positive impact on the motor skills of both girls and boys.

The results of the covariance analysis are shown in Table 5. After controlling for the pre-test scores of male and female students in each class during the second year of high school, the post-test scores of the experimental group were significantly higher than those of the control group. Specifically, in terms of passing and cutting coordination, male and female students in the experimental group showed significantly higher scores than those in the control group. Moreover, in basketball games, the post-test scores of male and female students in the experimental group were also significantly higher than those in the control group after controlling for their pre-test scores. These findings suggest that adopting the Chinese Healthy Physical Education Curriculum model can effectively enhance the sports skills of second-year high school students, in comparison with traditional physical education teaching methods.

**Table 5:** Results of the covariance analysis for motor skills between experimental and control group.

Variable	Group	Gender	*N*	*F*	*p*
1-minute shooting	Experimental Group	Girls	40	10.60	.002
	Control Group		38		
	Experimental Group	Boys	36	26.69	.001
	Control Group		35		
Passing and cutting coordination	Experimental Group	Girls	40	31.56	.001
	Control Group		38		
	Experimental Group	Boys	36	17.78	.001
	Control Group		35		
Game performance	Experimental Group	Girls	40	11.75	.001
	Control Group		38		
	Experimental Group	Boys	36	28.96	.001
	Control Group		35		

### 3.2 Physical fitness results

Table 6 presents the descriptive statistics of the physical fitness results, including the pre-test and post-test mean scores and standard deviations for each dimension, variable, gender, and group.

**Table 6:** Descriptive statistics for physical fitness results.

Dimension	Variabzle	Group	Gender	*N*	pre-test		post-test	
					*M*	*SD*	*M*	*SD*
Body composition	PBF (%)	Experimental Group	Girls	40	26.64	5.53	26.22	5.22
			Boys	36	20.70	7.87	19.82	7.40
		Control Group	Girls	38	27.11	6.18	27.20	5.91
			Boys	35	20.74	7.01	20.69	6.86
	WHR (%)	Experimental Group	Girls	40	0.75	0.05	0.75	0.05
			Boys	36	0.84	0.10	0.83	0.11
		Control Group	Girls	38	0.73	0.04	0.74	0.04
			Boys	35	0.82	0.06	0.83	0.05
Cardiopulmonary function	Vital Capacity (milliliter)	Experimental Group	Girls	40	3026.85	505.17	3142.20	502.90
			Boys	36	4432.08	795.28	4650.08	792.09
		Control Group	Girls	38	2941.00	502.57	2972.79	508.37
			Boys	35	4120.37	739.95	4150.94	759.21
	20-meter shuttle run (times)	Experimental Group	Girls	40	27.00	8.39	30.93	10.07
			Boys	36	47.39	18.38	54.92	20.61
		Control Group	Girls	38	26.61	8.36	26.50	8.35
			Boys	35	44.60	15.15	44.74	14.78
Muscle strength	Grip strength (kilogram)	Experimental Group	Girls	40	23.30	6.91	27.55	6.14
			Boys	36	47.69	13.30	50.61	12.71
		Control Group	Girls	38	21.47	8.49	23.22	9.06
			Boys	35	39.31	11.28	40.89	11.42
	Standing long jump (meter)	Experimental Group	Girls	40	1.57	0.20	1.65	0.19
			Boys	36	2.04	0.24	2.16	0.26
		Control Group	Girls	38	1.57	0.17	1.59	0.14
			Boys	35	2.06	0.21	2.12	0.23
Muscle endurance	1-minute crunches (times)	Experimental Group	Girls	40	36.85	9.82	40.80	9.05
			Boys	36	39.11	9.99	41.78	8.88
		Control Group	Girls	38	41.24	9.68	42.89	9.69
			Boys	35	39.94	6.02	41.14	5.63
Flexibility	Seated forward bend (centimeter)	Experimental Group	Girls	40	10.68	8.01	14.08	7.96
			Boys	36	7.94	10.07	9.89	9.33
		Control Group	Girls	38	6.16	4.81	8.42	5.67
			Boys	35	8.34	5.68	9.63	5.66
Speed	50-meter dash (second)	Experimental Group	Girls	40	8.66	0.73	8.52	0.67
			Boys	36	7.92	0.83	7.79	0.81
		Control Group	Girls	38	9.03	0.62	9.03	0.62
			Boys	35	7.56	0.46	7.53	0.44
Agility	20-secend lateral shuffles (times)	Experimental Group	Girls	40	30.28	4.43	35.20	3.72
			Boys	36	35.31	8.08	39.22	7.19
		Control Group	Girls	38	29.55	4.56	30.95	3.86
			Boys	35	25.03	6.50	26.91	5.61

In terms of body composition, the experimental class 1 for girls and boys’ experimental class 2 had a slightly lower post-test mean score for percent body fat (PBF) compared to their respective control classes, although the difference was minimal. A similar trend was observed in waist-to-hip ratio (WHR), where the girls’ experimental class 1 and boys’ experimental class 2 had slightly lower post-test mean scores compared to their respective control classes. Regarding cardiopulmonary function, both the girls’ experimental class 1 and boys’ experimental class 2 had higher post-test mean scores for vital capacity compared to their respective control classes. For the 20-meter shuttle run, both girls’ and boys’ experimental classes had higher post-test mean scores compared to their respective control classes. In terms of muscle strength, the girls’ experimental class 1 and boys’ experimental class 2 had higher post-test mean scores for grip strength compared to their respective control classes. However, for standing long jump, there was no significant difference observed between the experimental and control classes. For muscle endurance, the girls’ experimental class 1 and boys’ experimental class 2 had higher post-test mean scores for 1-minute crunches compared to their respective control classes. Regarding flexibility, the girls’ experimental class 1 and boys’ experimental class 2 had higher post-test mean scores for seated forward bend compared to their respective control classes. Finally, in speed and agility, the experimental classes had slightly better post-test mean scores compared to their respective control classes.

In summary, the experimental classes showed an overall improvement in physical fitness in most dimensions, variables, genders, and groups compared to the control classes. However, the differences were sometimes minimal and not significant, indicating a need for further investigation and analysis of the results to determine the effectiveness of the physical fitness intervention.

The results of the covariance analysis are shown in Table 7. Using pre-test scores as covariates, the study found that the post-test scores of male students in the experimental class were significantly lower than those of male students in the control class in terms of percent body fat (PBF), while there was no significant difference in PBF between female students in the experimental and control classes. The study also found that the experimental class had significantly lower waist-to-hip ratio (WHR) and significantly higher lung capacity, 20-meter shuttle run, grip strength, and standing long jump scores compared to the control class for both male and female students. Additionally, the experimental group had significantly higher scores on one-minute sit-ups and the 20-second shuttle run compared to the control group for both male and female students. However, the experimental group had significantly lower scores on the 50-meter run compared to the control group for both male and female students. The study concluded that using the Chinese Health and Physical Education Curriculum model can significantly improve physical fitness outcomes among male and female students in the second year of high school.

**Table 7:** Results of the covariance analysis for physical fitness between experimental and control group.

	Variable	Group	Gender	*N*	*F*	*p*
Body composition	PBF (%)	Experimental Group	Girls	40	1.41	.238
		Control Group		38		
		Experimental Group	Boys	36	6.11	.016
		Control Group		35		
	WHR (%)	Experimental Group	Girls	40	4.05	.048
		Control Group		38		
		Experimental Group	Boys	36	6.14	.016
		Control Group		35		
Cardiopulmonary function	Vital Capacity (milliliter)	Experimental Group	Girls	40	22.79	.001
		Control Group		38		
		Experimental Group	Boys	36	49.47	.001
		Control Group		35		
	20-meter shuttle run (times)	Experimental Group	Girls	40	25.75	.001
		Control Group		38		
		Experimental Group	Boys	36	62.84	.001
		Control Group		35		
Muscle strength	Grip strength (kilogram)	Experimental Group	Girls	40	24.39	.001
		Control Group		38		
		Experimental Group	Boys	36	5.25	.025
		Control Group		35		
	Standing long jump (meter)	Experimental Group	Girls	40	17.66	.001
		Control Group		38		
		Experimental Group	Boys	36	4.41	.040
		Control Group		35		
Muscle endurance	1-minute crunches (times)	Experimental Group	Girls	40	8.36	.005
		Control Group		38		
		Experimental Group	Boys	36	10.71	.002
		Control Group		35		
Flexibility	Seated forward bend (centimeter)	Experimental Group	Girls	40	4.29	.042
		Control Group		38		
		Experimental Group	Boys	36	1.02	.315
		Control Group		35		
Speed	50-meter dash (second)	Experimental Group	Girls	40	31.25	.001
		Control Group		38		
		Experimental Group	Boys	36	6.96	.010
		Control Group		35		
Agility	20-secend lateral shuffles (times)	Experimental Group	Girls	40	49.62	.001
		Control Group		38		
		Experimental Group	Boys	36	27.45	.001
		Control Group		35		

### 3.3 Motor cognitive ability results

Table 8 presents the descriptive statistics for the motor cognitive ability results, specifically for the variables of motor cognition and physical fitness awareness.

**Table 8:** Descriptive statistics for motor cognitive ability results.

Variable	Group	Gender	*N*	pre-test		post-test	
				*M*	*SD*	*M*	*SD*
Motor cognition	Experimental Group	Girls	40	18.63	4.88	27.13	2.05
		Boys	36	18.39	5.49	26.75	2.02
	Control Group	Girls	38	19.47	3.78	23.32	2.48
		Boys	35	20.86	3.31	24.11	2.47
Physical fitness awareness	Experimental Group	Girls	40	9.40	2.63	13.45	1.04
		Boys	36	9.47	2.63	13.47	1.16
	Control Group	Girls	38	9.24	2.40	11.32	1.93
		Boys	35	11.66	2.22	13.31	1.08

For the variable of motor cognition, the experimental class 1 for girls had a lower pre-test mean score compared to the control class 1, but this difference was not statistically significant. However, the experimental class 1 had a higher post-test mean score compared to the control class 1, and this difference was statistically significant. For boys, the experimental class 2 had a lower pre-test mean score compared to the control class 2, but this difference was not statistically significant. The experimental class 2 had a higher post-test mean score compared to the control class 2, and this difference was statistically significant.

For the variable of physical fitness awareness, the experimental class 1 for girls had a slightly higher pre-test mean score compared to the control class 1, but this difference was not statistically significant. The experimental class 1 had a significantly higher post-test mean score compared to the control class 1. For boys, the experimental class 2 had a lower pre-test mean score compared to the control class 2, but this difference was not statistically significant. The experimental class 2 had a significantly higher post-test mean score compared to the control class 2.

In summary, the experimental classes showed improvement in motor cognitive ability in terms of motor cognition and physical fitness awareness compared to the control classes. The differences were statistically significant for most comparisons, except for the pre-test mean scores in some cases. These results suggest that the physical fitness intervention had a positive impact on the motor cognition of the students. However, further research is needed to fully evaluate the effectiveness of the intervention.

Table 9 presents the results of a covariance analysis investigating the impact of gender and group membership on motor cognition and physical fitness awareness in experimental and control groups. The analysis revealed significant differences between the two groups in both variables. Girls in the experimental group scored higher in motor cognition and physical fitness awareness compared to girls in the control group. The same trend was observed for boys in the experimental group, who outperformed boys in the control group in both variables.

**Table 9:** Results of the covariance analysis for motor cognition between experimental and control group.

Variable	Group	Gender	*N*	*F*	*p*
Motor cognition	Experimental Group	Girls	40	35.56	.001
	Control Group	Girls	38		
	Experimental Group	Boys	36	98.62	.001
	Control Group	Boys	35		
Physical fitness awareness	Experimental Group	Girls	40	84.26	.001
	Control Group	Girls	38		
	Experimental Group	Boys	36	19.67	.001
	Control Group	Boys	35		

These results suggest that incorporating motor activities into the curriculum can positively affect motor cognition in students, particularly in motor cognition and physical fitness awareness. Gender differences were also observed, emphasizing the importance of considering gender when designing interventions to enhance motor cognition in school settings. These findings can inform future instructional approaches to promote motor cognitive abilities among students.

## 4 Discussion

### 4.1 Motor skill

The study measured motor skills using three tasks: 1-minute shooting, passing and cutting coordination, and game performance. The results show that both girls and boys in the experimental groups improved their mean scores from pre-test to post-test in all three tasks, and their improvements were greater than those in the control groups.

In the 1-minute shooting task, both the experimental and control groups showed improvement from pre-test to post-test. However, the experimental group had a larger improvement in mean scores compared to the control group. This suggests that the intervention program was effective in improving shooting accuracy for both girls and boys. It is important to note that while the girls in the experimental group had a higher mean score at post-test than the boys, the boys had a higher mean score at pre-test, indicating potential gender differences in initial motor skill levels. Similarly, in the passing and cutting coordination task, both the experimental and control groups showed improvement from pre-test to post-test, but the experimental group had a larger improvement in mean scores compared to the control group. This suggests that the intervention program was effective in improving passing and cutting coordination for both girls and boys. In the game performance task, both the experimental and control groups showed improvement from pre-test to post-test, with the experimental group showing a larger improvement in mean scores compared to the control group. This suggests that the intervention program was effective in improving game performance for both girls and boys [27, 28].

Overall, the study’s results suggest that the intervention program was effective in improving the motor skills of both girls and boys across all three tasks. The results align with previous research that has demonstrated the positive impact of intervention programs on motor skill development in children [29]. However, it is important to note that the study’s results are limited by the small sample size and the potential influence of other factors, such as natural maturation and practice effects.

### 4.2 Physical fitness

The findings of this study revealed that the experimental classes had an overall improvement in most variables of physical fitness compared to the control classes. The experimental classes demonstrated a slightly lower post-test mean score for PBF and WHR, indicating a trend towards healthier body composition. Moreover, the experimental classes exhibited higher post-test mean scores for vital capacity, shuttle run, grip strength, crunches, and seated forward bend, indicating improvement in cardiopulmonary function, muscle strength, muscle endurance, and flexibility, respectively. In terms of speed and agility, the experimental classes also showed a slight improvement in post-test mean scores.

The observed gender differences in initial motor skill levels in the 1-minute shooting task suggest that there may be a need to provide targeted intervention strategies for girls and boys to achieve optimal outcomes. Moreover, although the experimental classes showed an improvement in most dimensions of physical fitness, the differences were sometimes minimal and not significant, indicating a need for further investigation and analysis of the results. Therefore, it may be necessary to incorporate additional strategies or activities to enhance the effectiveness of physical fitness interventions for school-aged children [30, 31].

These findings are consistent with previous research that has shown the positive effects of physical fitness interventions on children’s physical fitness and health outcomes [32–34]. The results of this study can be used to inform the development of future physical fitness interventions that can be implemented in schools or community settings.

### 4.3 Motor cognitive ability

The study examined the impact of HPECM intervention on motor cognition and physical fitness awareness among students in two experimental classes compared to two control classes. The results of the study indicated that the experimental groups showed improvement in both motor cognition and physical fitness awareness compared to the control groups. The differences were statistically significant for most comparisons, except for the pre-test mean scores in some cases. These findings suggest that incorporating physical activity into the curriculum can positively impact motor cognition in students, particularly in motor cognition and physical fitness awareness.

The results of the study are consistent with previous research that has shown a positive relationship between physical activity and cognitive functioning. For instance, a study by Hillman found that regular physical activity can improve cognitive performance and brain function in children [35]. Similarly, a study by Diamond and Lee reported that physical activity can enhance cognitive control and working memory in children [36].

The study also highlighted the importance of considering gender when designing interventions to enhance motor cognition in school settings. The results showed that girls in the experimental group scored higher in motor cognition and physical fitness awareness compared to girls in the control group, and the same trend was observed for boys. These findings are consistent with previous research indicating that gender differences exist in physical activity levels and that girls tend to be less physically active than boys [37–39].

The study has some limitations. Firstly, the sample size was relatively small, and the study was conducted in a specific cultural and geographic context. Therefore, caution should be exercised when generalizing the results to other populations. Secondly, the research exclusively focused on basketball, lacking a comparative analysis with other activities or sports projects. Future research endeavors will include comparative studies across various projects. Additionally, the study did not investigate the long-term effects of the HPECM intervention on sports ability. Future research could address these limitations by conducting larger-scale studies in diverse populations and exploring the long-term effects of HPECM intervention on sports ability.

## 5. Conclusions

In conclusion, this study provides evidence that healthy physical education curriculum model can effectively promote sports ability in senior high school students. The structural characteristics of a healthy physical education curriculum model provide needed support for students learning, improve students’ sports ability, and can be an effective way to promote physical literacy in senior high school students. Further research is needed to explore the long-term effects of the healthy physical education curriculum model on sports ability in students.
