# Correction: An experimental study on the influence of healthy physical education curriculum model on sports ability of Chinese senior high school students

**DOI:** 10.1371/journal.pone.0336815

**Published:** 2025-11-13

**Authors:** The PLOS One Staff

All five authors, Shengting Dai, Qian Qiu, Yuancai Zhang, Jingfei Yan and Rongbin Yin, should not have been attributed equal contribution to this work.

The publisher apologizes for the error.
